# *tert*-Butyl *N*-(4-hy­droxy­benz­yl)-*N*-[4-(prop-2-yn­yloxy)benz­yl]carbamate

**DOI:** 10.1107/S160053681103683X

**Published:** 2011-09-14

**Authors:** Lei Ao, Jie-Hong Tu, Xuan Huang, Bao-Yue Ding

**Affiliations:** a College of Medicine, Jiaxing University, Jiaxing 314001, People’s Republic of China

## Abstract

In the crystal structure of the title compound, C_22_H_25_NO_4_, inter­molecular O—H⋯O hydrogen bonds involving the hy­droxy group of the 4-(amimometh­yl)phenol fragment and the C=O group connect the mol­ecules into infinite chains along the *c* axis. Two C atoms of the propyne group are disordered over two sites with occupancy factors of 0.53 (2) and 0.47 (2).

## Related literature

For applications of the title compound, see: Späth & König (2010 ▶); Juríček *et al.* (2011 ▶). For the synthesis of the title compound, see: Kim *et al.* (2004 ▶). For bond-length data, see: Allen *et al.* (1987 ▶).
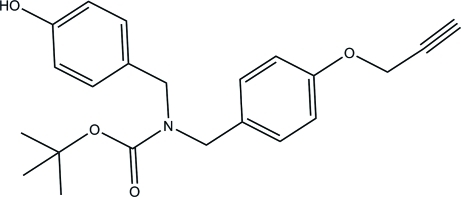


## Experimental

### 

#### Crystal data

C_22_H_25_NO_4_
*M* *_r_* = 367.43
Monoclinic, 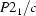

*a* = 18.6904 (8) Å
*b* = 6.2611 (4) Å
*c* = 17.3567 (7) Å
β = 96.791 (1)°
*V* = 2016.87 (18) Å^3^
*Z* = 4
Mo *K*α radiation
μ = 0.08 mm^−1^
*T* = 296 K
0.41 × 0.37 × 0.29 mm

#### Data collection

Rigaku R-AXIS RAPID/ZJUG diffractometer
Absorption correction: multi-scan (*ABSCOR*; Higashi, 1995 ▶) *T*_min_ = 0.957, *T*_max_ = 0.976
15741 measured reflections
3750 independent reflections
2099 reflections with *I* > 2σ(*I*)
*R*_int_ = 0.038

#### Refinement

*R*[*F* ^2^ > 2σ(*F* ^2^)] = 0.046
*wR*(*F* ^2^) = 0.149
*S* = 1.00
3750 reflections
268 parameters
4 restraints
H-atom parameters constrained
Δρ_max_ = 0.26 e Å^−3^
Δρ_min_ = −0.19 e Å^−3^

### 

Data collection: *RAPID-AUTO* (Rigaku, 1998 ▶); cell refinement: *RAPID-AUTO*; data reduction: *CrystalStructure* (Rigaku/MSC, 2002 ▶); program(s) used to solve structure: *SHELXS97* (Sheldrick, 2008 ▶); program(s) used to refine structure: *SHELXL97* (Sheldrick, 2008 ▶); molecular graphics: *ORTEP-3 for Windows* (Farrugia, 1997 ▶); software used to prepare material for publication: *WinGX* (Farrugia, 1999 ▶).

## Supplementary Material

Crystal structure: contains datablock(s) I, global. DOI: 10.1107/S160053681103683X/kj2185sup1.cif

Structure factors: contains datablock(s) I. DOI: 10.1107/S160053681103683X/kj2185Isup2.hkl

Supplementary material file. DOI: 10.1107/S160053681103683X/kj2185Isup3.cml

Additional supplementary materials:  crystallographic information; 3D view; checkCIF report

## Figures and Tables

**Table 1:** Hydrogen-bond geometry (Å, °)

*D*—H⋯*A*	*D*—H	H⋯*A*	*D*⋯*A*	*D*—H⋯*A*
O1—H1⋯O4^i^	0.82	1.94	2.745 (2)	167

Symmetry code: (i) 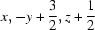.

**Fig. 1.**
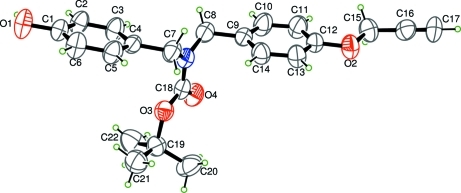
The molecular structure of title compound. Displacement ellipsoids are drawn at the 40% probability level. Only the major disorder component is shown.

**Fig. 2.**
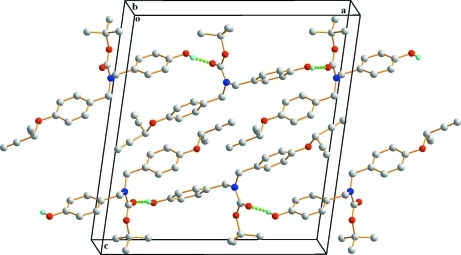
Crystal packing of the title compound, viewed down the b axis, showing the O—H···O the hydrogen bonds as green dashed lines. H atoms not involved in hydrogen bonding have been omitted. Both disorder compounds of the propyne group are shown.

## supplementary crystallographic information

### Comment

The amino group is one of the most important functional groups in molecules of biological relevance, of which histamine and dopamine are two representative examples. In the synthesis of amino-contaning compounds, the boc group is commonly used to protect the amino group when performing parallel chemical transformations (Späth *et al.* , 2010). The acetylene group, due to the presence of the carbon-carbon triple bond, is an ideal functional group for further postmodification by numerous synthetic transformations (Juríček *et al.* , 2011). In our exploration of structure-activity relationships of amino-contaning compounds, we recently obtained a crystal of an intermediate, which contains both a boc-protecting amino group and an acetylene group. We report its crystal structure here.

The molecular structure of the title compound is shown in Fig. 1. The dihedral angle of the rings C1—C6 and C9—C14 is 11.7 (3)°. The bond lengths and angles are within normal ranges (Allen *et al.*, 1987). The C18—N1 distance is 1.348 (3) Å, which is in the range of a typical double C=N bond. Atom O4 has a partial anionic character, as is shown by the lengthening of the C=O bond [1.226 (3) Å] compared to what is normally found for carbonyl groups. This atom acts as a hydrogen-bond acceptor for an intermolecular O—H···O hydrogen bond (Table 1). The hydrogen bonds are forming one-dimensional infinite chains along the *c* axis (Fig. 2).

### Experimental

The title compound was synthesized according to the method proposed by Kim *et al.* (2004). 4-(Amimomethyl)phenol (0.01 mol,1.23 g) and 4-(prop-2-gnyloxy)benzoldehyde were heated in anhydrous methanol for 2 h, then NaBH_4_ (0.1 mol,0.38 g) was added to the solution. The resulting solution was stirred for 30 minutes, then Boc_2_O (0.01 mol,2.18 g) was dropped into the solution. Colourless block-shaped single crystals suitable for X-ray structure determination were obtained by slow evaporation of the solution in a mixture of PE:EA(1:1,v:v). Yield: 51.7%.

### Refinement

Two C atoms of the propyne group are disordered over two sites. The occupancy factors refined to 0.53 (2) and 0.47 (2). H atoms were positioned geometrically and refined as riding groups, with O—H = 0.82 and C—H = 0.93 Å for aromatic H, 0.96 for methyl H, 0.97 for methylene H and constrained to ride on their parent atoms, with *U*iso(H) = *x U*_eq_(C), where *x* = 1.2 for aromatic and methylene H, *x* = 1.0 for H atoms bonded to the disorded C atoms of the propyne group and *x* = 1.5 for methyl H.

### Crystal data

**Table:**

C_22_H_25_NO_4_	*F*(000) = 784
*M**_r_* = 367.43	*D*_x_ = 1.210 Mg m^−^^3^
Monoclinic, *P*2_1_/*c*	Mo *K*α radiation, λ = 0.71073 Å
Hall symbol: -P 2ybc	Cell parameters from 9726 reflections
*a* = 18.6904 (8) Å	θ = 3.0–27.4°
*b* = 6.2611 (4) Å	µ = 0.08 mm^−^^1^
*c* = 17.3567 (7) Å	*T* = 296 K
β = 96.791 (1)°	Chunk, colorless
*V* = 2016.87 (18) Å^3^	0.41 × 0.37 × 0.29 mm
*Z* = 4	

### Data collection

**Table:**

Rigaku R-AXIS RAPID/ZJUG diffractometer	3750 independent reflections
Radiation source: rolling anode	2099 reflections with *I* > 2σ(*I*)
graphite	*R*_int_ = 0.038
Detector resolution: 10.00 pixels mm^-1^	θ_max_ = 25.5°, θ_min_ = 3.0°
ω scans	*h* = −22→22
Absorption correction: multi-scan (*ABSCOR*; Higashi, 1995)	*k* = −7→7
*T*_min_ = 0.957, *T*_max_ = 0.976	*l* = −21→19
15741 measured reflections	

### Refinement

**Table:**

Refinement on *F*^2^	Secondary atom site location: difference Fourier map
Least-squares matrix: full	Hydrogen site location: inferred from neighbouring sites
*R*[*F*^2^ > 2σ(*F*^2^)] = 0.046	H-atom parameters constrained
*wR*(*F*^2^) = 0.149	*w* = 1/[σ^2^(*F*_o_^2^) + (0.0635*P*)^2^ + 0.6687*P*] where *P* = (*F*_o_^2^ + 2*F*_c_^2^)/3
*S* = 1.00	(Δ/σ)_max_ < 0.001
3750 reflections	Δρ_max_ = 0.26 e Å^−^^3^
268 parameters	Δρ_min_ = −0.19 e Å^−^^3^
4 restraints	Extinction correction: *SHELXL97* (Sheldrick, 2008), Fc^*^=kFc[1+0.001xFc^2^λ^3^/sin(2θ)]^-1/4^
Primary atom site location: structure-invariant direct methods	Extinction coefficient: 0.0155 (18)

### Special details

**Table:**

Geometry. All e.s.d.'s (except the e.s.d. in the dihedral angle between two l.s. planes) are estimated using the full covariance matrix. The cell e.s.d.'s are taken into account individually in the estimation of e.s.d.'s in distances, angles and torsion angles; correlations between e.s.d.'s in cell parameters are only used when they are defined by crystal symmetry. An approximate (isotropic) treatment of cell e.s.d.'s is used for estimating e.s.d.'s involving l.s. planes.
Refinement. Refinement of *F*^2^ against ALL reflections. The weighted *R*-factor *wR* and goodness of fit *S* are based on *F*^2^, conventional *R*-factors *R* are based on *F*, with *F* set to zero for negative *F*^2^. The threshold expression of *F*^2^ > σ(*F*^2^) is used only for calculating *R*-factors(gt) *etc*. and is not relevant to the choice of reflections for refinement. *R*-factors based on *F*^2^ are statistically about twice as large as those based on *F*, and *R*- factors based on ALL data will be even larger.

### Fractional atomic coordinates and isotropic or equivalent isotropic displacement parameters (Å^2^)

**Table:**

	*x*	*y*	*z*	*U*_iso_*/*U*_eq_	Occ. (<1)
O1	0.17241 (12)	0.2936 (3)	0.76205 (9)	0.0818 (6)	
H1	0.1833	0.3985	0.7892	0.123*	
O2	0.43006 (9)	0.6806 (3)	0.13104 (10)	0.0726 (5)	
O3	0.13748 (8)	0.5836 (3)	0.40395 (8)	0.0592 (5)	
O4	0.19445 (10)	0.8841 (3)	0.36950 (9)	0.0695 (5)	
N1	0.25604 (11)	0.6051 (3)	0.42845 (10)	0.0605 (5)	
C1	0.19319 (13)	0.3250 (4)	0.68966 (12)	0.0564 (6)	
C2	0.22502 (14)	0.5106 (4)	0.66891 (13)	0.0643 (7)	
H2	0.2334	0.6214	0.7046	0.077*	
C3	0.24466 (15)	0.5329 (4)	0.59494 (13)	0.0664 (7)	
H3	0.2663	0.6593	0.5816	0.080*	
C4	0.23302 (11)	0.3722 (4)	0.54024 (12)	0.0513 (6)	
C5	0.20066 (14)	0.1883 (4)	0.56236 (13)	0.0648 (7)	
H5	0.1919	0.0776	0.5268	0.078*	
C6	0.18094 (15)	0.1640 (4)	0.63598 (14)	0.0712 (7)	
H6	0.1592	0.0378	0.6493	0.085*	
C7	0.25534 (14)	0.3881 (4)	0.45966 (13)	0.0633 (7)	
H7A	0.3032	0.3276	0.4604	0.076*	
H7B	0.2227	0.3019	0.4248	0.076*	
C8	0.32381 (13)	0.7155 (5)	0.42417 (13)	0.0712 (8)	
H8A	0.3601	0.6535	0.4622	0.085*	
H8B	0.3180	0.8639	0.4382	0.085*	
C9	0.35077 (12)	0.7072 (4)	0.34535 (12)	0.0584 (6)	
C10	0.39496 (15)	0.8655 (5)	0.32395 (15)	0.0776 (8)	
H10	0.4067	0.9780	0.3581	0.093*	
C11	0.42283 (15)	0.8647 (5)	0.25335 (15)	0.0762 (8)	
H11	0.4527	0.9746	0.2404	0.091*	
C12	0.40561 (12)	0.6991 (4)	0.20300 (13)	0.0579 (6)	
C13	0.36058 (13)	0.5388 (4)	0.22214 (14)	0.0649 (7)	
H13	0.3485	0.4271	0.1877	0.078*	
C14	0.33342 (13)	0.5442 (4)	0.29254 (14)	0.0652 (7)	
H14	0.3027	0.4359	0.3049	0.078*	
C15	0.48249 (14)	0.8306 (4)	0.11323 (15)	0.0756 (8)	
H15A	0.5194	0.8459	0.1571	0.091*	
H15	0.4601	0.9688	0.1024	0.091*	0.532 (4)
H15B	0.5046	0.7474	0.0752	0.091*	0.468 (4)
C16A	0.5155 (2)	0.7544 (7)	0.0443 (2)	0.0661 (14)	0.532 (4)
C17A	0.5407 (3)	0.6902 (8)	−0.0069 (2)	0.0760 (17)	0.532 (4)
H17A	0.5615	0.6372	−0.0491	0.091*	0.532 (4)
C16B	0.4509 (3)	1.0432 (7)	0.0868 (3)	0.0689 (17)	0.468 (4)
C17B	0.4307 (4)	1.2108 (7)	0.0727 (4)	0.086 (2)	0.468 (4)
H17B	0.4141	1.3484	0.0611	0.103*	0.468 (4)
C18	0.19532 (13)	0.7046 (4)	0.39779 (12)	0.0566 (6)	
C19	0.06391 (13)	0.6547 (4)	0.37645 (14)	0.0623 (6)	
C20	0.05670 (17)	0.6953 (5)	0.28996 (16)	0.0932 (10)	
H20A	0.0806	0.8265	0.2800	0.140*	
H20B	0.0066	0.7054	0.2703	0.140*	
H20C	0.0783	0.5797	0.2647	0.140*	
C21	0.01977 (15)	0.4612 (5)	0.39359 (16)	0.0809 (8)	
H21A	0.0320	0.3431	0.3624	0.121*	
H21B	−0.0306	0.4932	0.3818	0.121*	
H21C	0.0299	0.4245	0.4475	0.121*	
C22	0.04566 (18)	0.8454 (5)	0.4239 (2)	0.1049 (11)	
H22A	0.0587	0.8160	0.4780	0.157*	
H22B	−0.0051	0.8734	0.4144	0.157*	
H22C	0.0718	0.9680	0.4093	0.157*	

### Atomic displacement parameters (Å^2^)

**Table:**

	*U*^11^	*U*^22^	*U*^33^	*U*^12^	*U*^13^	*U*^23^
O1	0.1286 (16)	0.0695 (13)	0.0529 (10)	−0.0072 (12)	0.0346 (10)	0.0001 (9)
O2	0.0827 (12)	0.0748 (13)	0.0651 (11)	−0.0114 (10)	0.0292 (9)	−0.0031 (9)
O3	0.0653 (10)	0.0561 (10)	0.0566 (9)	0.0006 (8)	0.0088 (7)	0.0070 (8)
O4	0.0927 (13)	0.0557 (11)	0.0608 (10)	−0.0063 (9)	0.0113 (9)	0.0095 (8)
N1	0.0651 (12)	0.0708 (14)	0.0463 (10)	0.0014 (11)	0.0101 (9)	0.0085 (10)
C1	0.0717 (15)	0.0532 (15)	0.0454 (12)	0.0012 (12)	0.0109 (11)	0.0046 (11)
C2	0.0915 (18)	0.0562 (16)	0.0455 (13)	−0.0079 (14)	0.0090 (12)	−0.0060 (11)
C3	0.0921 (18)	0.0566 (16)	0.0522 (14)	−0.0155 (13)	0.0155 (13)	−0.0014 (12)
C4	0.0559 (13)	0.0566 (15)	0.0412 (11)	0.0052 (11)	0.0046 (9)	0.0014 (10)
C5	0.0825 (17)	0.0591 (16)	0.0529 (14)	−0.0081 (13)	0.0089 (12)	−0.0107 (12)
C6	0.102 (2)	0.0533 (16)	0.0612 (15)	−0.0136 (14)	0.0206 (14)	−0.0054 (12)
C7	0.0765 (16)	0.0657 (17)	0.0485 (13)	0.0155 (13)	0.0105 (11)	0.0065 (11)
C8	0.0694 (16)	0.093 (2)	0.0519 (13)	−0.0119 (15)	0.0077 (12)	−0.0025 (13)
C9	0.0558 (13)	0.0720 (17)	0.0475 (12)	−0.0059 (12)	0.0061 (10)	0.0016 (12)
C10	0.0908 (19)	0.087 (2)	0.0562 (15)	−0.0329 (17)	0.0127 (14)	−0.0139 (14)
C11	0.0831 (18)	0.084 (2)	0.0631 (16)	−0.0333 (16)	0.0160 (13)	−0.0054 (14)
C12	0.0586 (13)	0.0661 (16)	0.0506 (13)	0.0006 (12)	0.0131 (11)	0.0036 (12)
C13	0.0726 (16)	0.0621 (16)	0.0632 (15)	−0.0091 (13)	0.0215 (12)	−0.0066 (12)
C14	0.0676 (15)	0.0668 (17)	0.0636 (15)	−0.0105 (13)	0.0179 (12)	0.0011 (13)
C15	0.0773 (18)	0.078 (2)	0.0759 (17)	−0.0071 (15)	0.0281 (14)	0.0091 (15)
C16A	0.074 (3)	0.065 (3)	0.059 (3)	−0.008 (3)	0.006 (2)	0.015 (2)
C17A	0.099 (4)	0.082 (4)	0.052 (3)	0.004 (3)	0.032 (3)	0.003 (3)
C16B	0.078 (4)	0.074 (4)	0.058 (3)	−0.014 (3)	0.022 (3)	−0.009 (3)
C17B	0.124 (6)	0.056 (4)	0.083 (4)	−0.005 (4)	0.030 (4)	−0.002 (3)
C18	0.0739 (16)	0.0567 (16)	0.0407 (12)	−0.0044 (13)	0.0126 (11)	−0.0007 (11)
C19	0.0630 (14)	0.0604 (16)	0.0641 (14)	0.0043 (12)	0.0096 (12)	−0.0031 (12)
C20	0.100 (2)	0.100 (2)	0.0728 (17)	−0.0091 (19)	−0.0149 (16)	0.0252 (17)
C21	0.0818 (18)	0.080 (2)	0.0820 (19)	−0.0154 (16)	0.0134 (15)	0.0000 (15)
C22	0.091 (2)	0.078 (2)	0.150 (3)	0.0079 (17)	0.034 (2)	−0.037 (2)

### Geometric parameters (Å, °)

**Table:**

O1—C1	1.372 (3)	C10—H10	0.9300
O1—H1	0.8200	C11—C12	1.369 (3)
O2—C12	1.385 (3)	C11—H11	0.9300
O2—C15	1.417 (3)	C12—C13	1.376 (3)
O3—C18	1.335 (3)	C13—C14	1.378 (3)
O3—C19	1.470 (3)	C13—H13	0.9300
O4—C18	1.226 (3)	C14—H14	0.9300
N1—C18	1.348 (3)	C15—C16A	1.488 (3)
N1—C8	1.452 (3)	C15—C16B	1.506 (3)
N1—C7	1.464 (3)	C15—H15A	0.9700
C1—C2	1.373 (3)	C15—H15	0.9700
C1—C6	1.374 (3)	C15—H15B	0.9700
C2—C3	1.383 (3)	C16A—C17A	1.128 (3)
C2—H2	0.9300	C16A—H15B	0.5978
C3—C4	1.383 (3)	C17A—H17A	0.9300
C3—H3	0.9300	C16B—C17B	1.132 (3)
C4—C5	1.376 (3)	C17B—H17B	0.9300
C4—C7	1.509 (3)	C19—C22	1.512 (4)
C5—C6	1.379 (3)	C19—C20	1.513 (4)
C5—H5	0.9300	C19—C21	1.515 (4)
C6—H6	0.9300	C20—H20A	0.9600
C7—H7A	0.9700	C20—H20B	0.9600
C7—H7B	0.9700	C20—H20C	0.9600
C8—C9	1.514 (3)	C21—H21A	0.9600
C8—H8A	0.9700	C21—H21B	0.9600
C8—H8B	0.9700	C21—H21C	0.9600
C9—C10	1.369 (3)	C22—H22A	0.9600
C9—C14	1.384 (3)	C22—H22B	0.9600
C10—C11	1.387 (3)	C22—H22C	0.9600
			
C1—O1—H1	109.5	C14—C13—H13	120.1
C12—O2—C15	116.86 (19)	C13—C14—C9	121.6 (2)
C18—O3—C19	122.50 (19)	C13—C14—H14	119.2
C18—N1—C8	117.3 (2)	C9—C14—H14	119.2
C18—N1—C7	122.1 (2)	O2—C15—C16A	109.0 (3)
C8—N1—C7	120.4 (2)	O2—C15—C16B	113.3 (3)
C2—C1—O1	122.7 (2)	C16A—C15—C16B	102.9 (3)
C2—C1—C6	119.1 (2)	O2—C15—H15A	109.9
O1—C1—C6	118.3 (2)	C16A—C15—H15A	109.9
C1—C2—C3	120.0 (2)	C16B—C15—H15A	111.6
C1—C2—H2	120.0	O2—C15—H15	109.9
C3—C2—H2	120.0	C16A—C15—H15	109.9
C4—C3—C2	121.8 (2)	H15A—C15—H15	108.3
C4—C3—H3	119.1	O2—C15—H15B	98.9
C2—C3—H3	119.1	C16B—C15—H15B	116.8
C5—C4—C3	117.1 (2)	H15A—C15—H15B	105.5
C5—C4—C7	119.5 (2)	H15—C15—H15B	123.8
C3—C4—C7	123.4 (2)	C17A—C16A—C15	177.7 (5)
C4—C5—C6	121.7 (2)	C17A—C16A—H15B	154.1
C4—C5—H5	119.2	C16A—C17A—H17A	180.0
C6—C5—H5	119.2	C17B—C16B—C15	173.8 (6)
C1—C6—C5	120.4 (2)	C16B—C17B—H17B	180.0
C1—C6—H6	119.8	O4—C18—O3	125.5 (2)
C5—C6—H6	119.8	O4—C18—N1	123.5 (2)
N1—C7—C4	114.8 (2)	O3—C18—N1	111.0 (2)
N1—C7—H7A	108.6	O3—C19—C22	109.0 (2)
C4—C7—H7A	108.6	O3—C19—C20	110.1 (2)
N1—C7—H7B	108.6	C22—C19—C20	114.1 (3)
C4—C7—H7B	108.6	O3—C19—C21	101.8 (2)
H7A—C7—H7B	107.5	C22—C19—C21	111.2 (2)
N1—C8—C9	114.55 (19)	C20—C19—C21	110.0 (2)
N1—C8—H8A	108.6	C19—C20—H20A	109.5
C9—C8—H8A	108.6	C19—C20—H20B	109.5
N1—C8—H8B	108.6	H20A—C20—H20B	109.5
C9—C8—H8B	108.6	C19—C20—H20C	109.5
H8A—C8—H8B	107.6	H20A—C20—H20C	109.5
C10—C9—C14	117.1 (2)	H20B—C20—H20C	109.5
C10—C9—C8	119.8 (2)	C19—C21—H21A	109.5
C14—C9—C8	123.1 (2)	C19—C21—H21B	109.5
C9—C10—C11	122.6 (2)	H21A—C21—H21B	109.5
C9—C10—H10	118.7	C19—C21—H21C	109.5
C11—C10—H10	118.7	H21A—C21—H21C	109.5
C12—C11—C10	118.8 (2)	H21B—C21—H21C	109.5
C12—C11—H11	120.6	C19—C22—H22A	109.5
C10—C11—H11	120.6	C19—C22—H22B	109.5
C11—C12—C13	120.2 (2)	H22A—C22—H22B	109.5
C11—C12—O2	124.1 (2)	C19—C22—H22C	109.5
C13—C12—O2	115.7 (2)	H22A—C22—H22C	109.5
C12—C13—C14	119.7 (2)	H22B—C22—H22C	109.5
C12—C13—H13	120.1		
			
O1—C1—C2—C3	−179.9 (2)	C10—C11—C12—C13	0.9 (4)
C6—C1—C2—C3	−0.4 (4)	C10—C11—C12—O2	−180.0 (2)
C1—C2—C3—C4	0.1 (4)	C15—O2—C12—C11	7.5 (4)
C2—C3—C4—C5	0.2 (4)	C15—O2—C12—C13	−173.4 (2)
C2—C3—C4—C7	−178.4 (2)	C11—C12—C13—C14	−0.6 (4)
C3—C4—C5—C6	−0.4 (4)	O2—C12—C13—C14	−179.8 (2)
C7—C4—C5—C6	178.3 (2)	C12—C13—C14—C9	−0.5 (4)
C2—C1—C6—C5	0.3 (4)	C10—C9—C14—C13	1.3 (4)
O1—C1—C6—C5	179.8 (2)	C8—C9—C14—C13	−178.2 (2)
C4—C5—C6—C1	0.1 (4)	C12—O2—C15—C16A	165.7 (3)
C18—N1—C7—C4	−79.6 (3)	C12—O2—C15—C16B	−80.4 (3)
C8—N1—C7—C4	105.1 (2)	C19—O3—C18—O4	0.1 (3)
C5—C4—C7—N1	151.9 (2)	C19—O3—C18—N1	178.88 (18)
C3—C4—C7—N1	−29.5 (3)	C8—N1—C18—O4	−2.7 (3)
C18—N1—C8—C9	−77.4 (3)	C7—N1—C18—O4	−178.1 (2)
C7—N1—C8—C9	98.1 (3)	C8—N1—C18—O3	178.44 (18)
N1—C8—C9—C10	154.0 (3)	C7—N1—C18—O3	3.0 (3)
N1—C8—C9—C14	−26.5 (4)	C18—O3—C19—C22	−64.6 (3)
C14—C9—C10—C11	−1.1 (4)	C18—O3—C19—C20	61.2 (3)
C8—C9—C10—C11	178.5 (3)	C18—O3—C19—C21	177.9 (2)
C9—C10—C11—C12	−0.1 (4)		

### Hydrogen-bond geometry (Å, °)

**Table:**

*D*—H···*A*	*D*—H	H···*A*	*D*···*A*	*D*—H···*A*
O1—H1···O4^i^	0.82	1.94	2.745 (2)	167

Symmetry codes: (i) *x*, −*y*+3/2, *z*+1/2.
